# Possible Role of Hyperinsulinemia and Insulin Resistance in Lower Vitamin D Levels in Overweight and Obese Patients

**DOI:** 10.1155/2013/921348

**Published:** 2013-01-14

**Authors:** Giovanni De Pergola, Alessandro Nitti, Nicola Bartolomeo, Antonella Gesuita, Vito Angelo Giagulli, Vincenzo Triggiani, Edoardo Guastamacchia, Franco Silvestris

**Affiliations:** ^1^Clinical Nutrition Unit, Department of Biomedical Sciences and Human Oncology, Section of Clinical Oncology, School of Medicine, University of Bari, Policlinico, Piazza Giulio Cesare 11, 70124 Bari, Italy; ^2^Department of Biomedical Sciences and Human Oncology, School of Medicine, University of Bari, Policlinico, Piazza Giulio Cesare, 70124 Bari, Italy; ^3^Outpatient Clinic for Metabolic Diseases and Endocrinology, Conversano Hospital, ASL Bari, 70014 Conversano, Italy; ^4^Section of Endocrinology and Metabolic Diseases, Department of Emergency and Organ Transplantation, School of Medicine, University of Bari, Policlinico, Piazza Giulio Cesare, 70124 Bari, Italy

## Abstract

A cohort of 66 healthy overweight and obese patients, 53 women and 13 men were examined. Waist circumference and fasting 25(OH)D, insulin, glucose, lipid (cholesterol, HDL cholesterol, and triglyceride), C-reactive protein (CRP), and complement 3 (C_3_), and 4 (C_4_) serum concentrations were measured. Insulin resistance was assessed by the homeostasis model assessment (HOMA_IR_). *Results.* 25(OH)D levels showed a significant negative correlation with BMI (*P* < 0.01), waist circumference (*P* < 0.05), fasting insulin (*P* < 0.01), HOMA_IR_ (*P* < 0.01), triglycerides (*P* < 0.01), CRP (*P* < 0.01), C_3_ (*P* < 0.05), and C_4_ (*P* < 0.05). Multiple regression analyses were performed with 25(OH)D as the dependent variable and BMI (or waist circumferences), fasting insulin (or HOMA_IR_), triglycerides, and CRP (or C_3_ or C_4_) as independent variables. Only insulin or HOMA_IR_ maintained a significant independent association with 25(OH)D levels, whereas vitamin D did not maintain a significant independent association with CRP or C_3_ or C_4_ concentrations. *Conclusions.* The present study, performed in overweight and obese subjects, shows that 25(OH)D levels are negatively associated with inflammatory parameters such as CRP and C_3_ and C_4_ levels, but not independently of BMI, body fat distribution, insulin levels, or insulin resistance. Our results suggest that hyperinsulinemia and/or insulin resistance are directly responsible for decrease of 25(OH)D levels in obesity.

## 1. Introduction

Vitamin D is well known to be involved in the calcium and bone metabolism, and most observational and randomized placebo-controlled trials concerning this vitamin have focused on the skeletal effects and have linked low vitamin D levels to fractures [1–3]. However, interest is growing in the nonskeletal effects of vitamin D [4] and in the relationship between vitamin D deficiency and diseases such as obesity, diabetes mellitus, and metabolic syndrome.

Serum 25-hydroxyvitamin D3 (25(OH)D) level reflects total vitamin D from dietary intake and sunlight exposure, as well as the conversion of vitamin D from adipose stores in the liver. Therefore, it is the best indicator of overall vitamin D status [5].

Several studies have demonstrated that serum 25(OH)D concentrations are inversely correlated with measures of obesity such as body mass index (BMI), waist circumference, and subcutaneous and visceral fat [6–10].

Insulin resistance and hyperinsulinemia are typical features of abdominal obesity, and, interestingly, inverse associations between 25(OH)D levels and fasting insulin concentrations [11–13] or insulin resistance (measured by HOMA_IR_ index) [11–13] or insulin sensitivity index [14, 15] have been reported in most of the studies, even independently of BMI or obesity [13].

Some cross-sectional studies indicate that low vitamin D is also associated with higher serum levels of inflammatory biomarkers, such as C-reactive protein (CRP), interleukin-6 (IL-6), and tumor necrosis factor *α* (TNF-*α*) in either healthy [16–19] or obese subjects [20]. However, the relationship between vitamin D and inflammation is not fully clear. In fact, other studies have not confirmed an inverse relationship between vitamin D and inflammation parameters in normal weight [21, 22] and obese patients [23]. Moreover, when healthy overweight subjects participating in a weight-reduction program were supplemented with vitamin D (3332 IU cholecalciferol/day for twelve months), they did not show a decrease in serum CRP and IL-6 and concentration [24]. Lastly, the National Health and Nutrition Examination Survey (2001 to 2006) observed a significant independent inverse relation between 25(OH)D and CRP levels only when 25(OH)D concentrations were lower than 21 ng/mL [25].

Obesity is characterized by a condition of low-grade chronic inflammation, and it is well known that adipocytes express TNF-*α* and may produce 30%–40% of circulating levels of IL-6 [26], the main regulator of CRP production in the liver. It is noteworthy that low-grade chronic inflammation is responsible for the activation of the complement system, a situation that could contribute to the metabolic complications observed in obesity. No study has investigated the relationship between 25(OH)D and complement protein circulating levels. 

The complement is a system of proteins functionally interacting with each other in order to provide many of the effector functions of humoral immunity and inflammation. The central component of the complement system is the C_3_ fraction, as all the pathways for the activation of the system converge there. Interestingly, adipocytes are an important source of C_3_ production, in addition to that produced in the liver and in activated macrophages. Adipose tissue also produces all the factors of the alternative pathway for the complement activation [27], and human adipocytes express the genes that code the proteins activating the complement system [28]. A positive correlation was found between baseline insulin levels and C_3_ levels [29], and serum C_3_ has been considered as an inflammatory marker of insulin resistance [30]. Moreover, we recently found a direct association between waist circumference and C_3_ serum concentrations in overweight and obese patients, independently of insulin resistance [31].

To the best of our knowledge, no study has simultaneously evaluated vitamin D, insulin, insulin resistance, CRP, and C_3_ and C_4_ serum levels in a population of healthy obese subjects. Therefore, the present study was addressed to examine whether CRP and C_3_ and C_4_ serum levels are associated with vitamin D concentrations in overweight and obese patients, independently of BMI, body fat distribution, and insulin levels, all parameters well known to influence vitamin D concentrations. To this aim, a cohort of 66 overweight and obese patients, aged 18–55 years, were investigated.

## 2. Methods

### 2.1. Subject Population

The patients were consecutively enrolled at the Outpatient Clinic of Clinical Nutrition, Section of Clinical Oncology, Department of Internal Medicine and Clinical Oncology, University of Bari, School of Medicine. 

Concerning the inclusion criteria, subjects were recruited at the first medical examination whether they showed a BMI higher than 25.0 and were not using any drug (including oral contraceptives for premenopausal women and hormone replacement therapy for postmenopausal women). They came to the Outpatient Clinic with the aim of losing weight and/or to have dietetic and lifestyle suggestions. Subjects were defined overweight whether they had a BMI between 25.0 and 29.9 kg/m^2^ and obese whether they had a BMI ≥ 30 kg/m^2^. 

Patients with the following disorders were excluded: endocrinological diseases, diabetes mellitus, chronic inflammatory diseases, stable hypertension, angina pectoris, heart infarction, congenital heart disease, stroke, and transient ischemic attack. Thus, the study enrolled 66 patients, including 53 women and 13 men, aged 18–55 years. 

All subjects gave their informed consent for the study, which was performed in accordance with the guidelines proposed in the Declaration of Helsinki. 

All patients had fasting blood glucose levels lower than 126 mg/dL, and they were judged in good health on the basis of physical examination, medical history, routine blood work, urinalysis, and electrocardiogram. None of the patients was on hypocaloric diet or had been involved into intensive or competitive physical activity prior to the enrollment. During the testing period, all subjects were asked to keep their normal mixed diet and not to perform any sporting activity. The day before the measurement, they were abstained from both caffeinated and alcoholic drinks but maintained their normal diet.

#### 2.1.1. Anthropometric Measurements and General Data

Body weight was measured to the nearest kg. Height was determined to the nearest cm. BMI was calculated as the weight (kg) divided by the square of height (m). Waist circumference was measured at the narrowest part of the abdomen, that is, at the natural indentation between the 10th rib and the iliac crest (minimum waist).

#### 2.1.2. Hormone and Metabolic Parameters

Blood samples were drawn between 08:00 h and 09:00 h after an overnight fast. Serum insulin concentrations were measured by radioimmunoassay (Behring, Scoppito, Italy), and intra- and interassay coefficients of variation were 3.7% and 7.5%, respectively. Plasma 25(OH)D was quantified by a chemiluminescence method (Diasorin Inc., Stillwater, USA), and all samples were analyzed in duplicate. The assay had intraclass CV of 5.3%–6.7% and interclass CV of 4.6%–8.7%.

The C_3_ and C_4_ fractions of the complement were determined by immunonephelometry (Behring Nephelometer II (BNII); Dade-Behring, Marburg, Germany). Intra- and interassay coefficients of variation were lower than 6%. CRP was measured by a particle-enhanced turbidimetric immunoassay (PETIA) technique (Siemens Healthcare Diagnostic Inc., Newark, DE, USA). Intra- and interassay coefficients of variation were lower than 8%. It is noteworthy that this assay meets the recommendations of the American Heart Association and the Centers for Disease Control (2003) for determining patients at high risk for cardiovascular disease [32].

Plasma glucose levels were determined by the glucose-oxidase method (Sclavo, Siena, Italy). Plasma lipids (triglycerides, total cholesterol, and HDL cholesterol) were determined by an automatic colorimetric method (Hitachi; Boehringer Mannheim, Mannheim, Germany). 

Insulin resistance was assessed by using the homeostasis model assessment (HOMA_IR_) [33]. 

### 2.2. Statistics

The sample characteristics were expressed by mean and standard deviation of the measured parameters. Variables with a skewed distribution were transformed before any statistical analyses, to improve the approximation to a Gaussian distribution: insulin, HOMA_IR_, BMI, triglycerides, CRP, and C_4_ (positively skewed) were transformed into their logarithms, while C_3_ (negatively skewed) was elevated to the square. Several univariate linear regression analyses were performed to test the joint effect of different variables on plasma 25(OH)D levels, and, for each model, the assumptions of linearity, the normality of the residuals (Shapiro-Wilk test), the homoskedasticity (Breusch-Pagan test), and the absence of serial correlation (Durbin-Watson value) were verified; the measures of relationships were also evaluated by Pearson's correlation coefficient. The variables having a *P* value <0.05 in univariate *t*-test were selected for inclusion in multivariate linear regression model analyses. Concerning the power analysis, based on Type III *F* test of one predictor adjusting for the other three predictors (excluding the intercept) in a regression model with a significance level of 0.05, assuming an unconditional model with normal predictors, a sample size of 66 (i.e., the number of patients examined in the present study) has a power of 0.797. Statistical and power analyses were performed using SAS software Version 9.3 for PC.

## 3. Results

Table 1 shows the general, anthropometric, hormone, and metabolic parameters in the study population.

Table 2 shows the relationship between 25(OH)D levels and general, anthropometric, hormone, and metabolic parameters. Variables significantly predictive of 25(OH)D levels in univariate linear regression models were BMI, waist circumference, fasting insulin, HOMA_IR_, triglycerides, CRP, C_3_ and C_4_.

Concerning multiple regression analyses, a first analysis was performed by considering 25(OH)D as the dependent variable and BMI, fasting CRP, insulin (or HOMA_IR_), and triglycerides as independent variables (fitted model: *F* = 5.81, *P* < 0.001, adjusted *R* ^2^ = 0.237), and 25(OH)D did not maintain an independent association with CRP (*β* = −1.685, *P* = 0.136). A second multiple regression analysis was performed by considering 25(OH)D as the dependent variable and fasting C_3_, BMI, insulin (or HOMA_IR_), and triglycerides as independent variables (fitted model: *F* = 5.73, *P* < 0.001, adjusted *R* ^2^ = 0.240), and 25(OH)D did not maintain an independent association with C_3_ (*β* = −2.702, *P* = 0.158). A third multiple regression analysis was performed by considering 25(OH)D as the dependent variable and fasting C_4_, BMI, insulin (or HOMA_IR_), and triglycerides as independent variables (fitted model: *F* = 5.06, *P* = 0.001, adjusted *R* ^2^ = 0.213), and 25(OH)D did not maintain an independent association with C_4_ (*β* = −1.108, *P* = 0.755). 25(OH)D maintained a significant independent association only with insulin (*P* < 0.05) or HOMA_IR_ (*P* < 0.05), but not with BMI and triglycerides, in all of these multiple regressions. 

## 4. Discussion

At the best of our knowledge, this is the first study examining the relationship between complement factors and vitamin D levels in apparently healthy overweight and obese subjects. 

We show that serum vitamin D levels are negatively related to C_3_ and C_4_ concentrations, but these correlations were not maintained after adjustment for BMI, insulin levels, and insulin resistance. Seemingly, as previously shown in severely obese patients [20], vitamin D and CRP levels were inversely associated, but also this correlation was not maintained after adjustment for BMI, insulin, and insulin resistance, suggesting that the negative association between vitamin D and investigated inflammation parameters (CRP and C_3_ and C_4_) is only indirectly mediated by factors associated with obesity. 

According to previous studies [4–10], we found an inverse correlation between vitamin D levels and BMI (or waist circumference). As far as the mechanism why obese individuals have lower serum 25(OH)D concentrations, 25(OH)D has a high lipid-solubility, and it may well be that this vitamin is under sequestration when adipose tissue is in excess, resulting in reduced 25(OH)D bioavailability [34]. In line with this hypothesis, weight loss of 10%, obtained by a 20-week low-calorie diet, increased 25(OH)D levels in obese women [35]. Another study confirmed that serum vitamin D levels were higher after weight loss induced by a 2-year dietary intervention [36]. An alternate explanation is that higher leptin and IL-6 circulating levels, mostly secreted by adipose tissue, may have inhibitory effects on 25(OH)D synthesis via their receptors [37]. 

According to previous studies [11–16], we also found a negative correlation between vitamin D levels and insulin levels and insulin resistance (measured by HOMA_IR_). It is noteworthy that insulin or HOMA_IR_ was the only parameter maintaining an inverse relationship with vitamin D levels independently of other variables significantly correlated with vitamin D in this study (BMI, waist circumference, triglycerides, CRP, and C_3_ and C_4_). 

These results suggest that hyperinsulinemia and insulin resistance drive the negative association between serum vitamin D and inflammation parameters (CRP, and C_3_ and C_4_) in obesity. 

This is the first study raising the possibility that higher insulin levels and/or insulin resistance are directly responsible for lower vitamin D levels, but we do not have our own data to explain this result, and this is the limitation of this study. How hyperinsulinemia can cause a decrease in plasma vitamin D concentration needs to be addressed further, and this may be the direction of future studies in the obesity research area. However, it may well be that hyperinsulinemia *per se* or nonalcoholic fatty liver disease (NAFLD) associated with insulin resistance inhibits the activity of liver 25-hydroxylase, thus explaining lower production and circulating levels of 25(OH)D in obesity.

Interestingly, since low vitamin D is a risk factor for diabetes mellitus [38], it may well be that the effect of hyperinsulinemia on vitamin D levels may be one of the mechanisms that through insulin resistance enhances the risk of diabetes.

How low plasma vitamin D levels can change insulin concentration and sensitivity has been shown. In fact, vitamin D increases both insulin secretion [39, 40] and insulin sensitivity. Concerning insulin secretion, vitamin D receptors are expressed in both pancreatic *β*-cells and skeletal muscle cells, and vitamin D supplementation results in increased insulin release and insulin-mediated glucose transport [41]. Concerning the possible influence of vitamin D on insulin effect, two randomized controlled trials of high-dose vitamin D supplementation showed a significant increase in insulin sensitivity in insulin-resistant Indian men or South-Asian women, either after 6 weeks or 6 months of treatment, respectively [42, 43]. Interestingly, in vitro studies have even shown that vitamin D may influence insulin sensitivity via a direct stimulatory effect on insulin receptor expression [44].

In conclusion, the present study shows for the first time that vitamin D is negatively related to complement factor (C_3_ and C_4_) levels. These correlations were not maintained after adjustment for BMI (or waist circumference), insulin (or HOMA_IR_), and triglyceride levels, and, in particular, only insulin and HOMA_IR_ maintained an inverse relationship with vitamin D levels independently of other variables. Therefore, our study strongly suggests that hyperinsulinemia and insulin resistance are the main factors responsible for decrease of vitamin D concentrations in obese patients.

## Figures and Tables

**Table 1:** General, anthropometric, hormone, and metabolic parameters in subjects under study (*n* = 66).

Age (years)	36.3 ± 11.7
Body mass index (kg·m^−2^)	31.7 ± 5.0
Waist circumference (cm)	103.7 ± 12.6
Systolic blood pressure (mmHg)	128.3 ± 15.1
Diastolic blood pressure (mmHg)	86.2 ± 10.4
25(OH)D (ng/mL)	18.2 ± 7.7
Fasting blood glucose (mg/dL)	90.3 ± 8.4
Fasting insulin (*μ*UI/mL)	18.7 ± 8.1
HOMA_IR_	4.20 ± 1.98
Triglycerides (mg/mL)	90.1 ± 51.3
HDL cholesterol (mg/mL)	50.8 ± 13.0
Total cholesterol (mg/mL)	187.0 ± 42.5
CRP (mg/dL)	0.48 ± 0.46
C_3_ (g/L)	1.18 ± 0.22
C_4_ (g/L)	0.27 ± 0.08

HOMA_IR_: homeostasis model assessment.

**Table 2:** Pearson correlation coefficients of 25(OH)D levels with general, anthropometric, hormone, and metabolic parameters in subjects under study (*n* = 66).

	25(OH)D (ng/mL)
Age (years)	−0.118
Body mass index (kg·m^−2^)	−0.346**
Waist circumference (cm)	−0.296*
Systolic blood pressure (mmHg)	−0.120
Diastolic blood pressure (mmHg)	−0.137
Fasting blood glucose (mg/dL)	−0.149
Fasting insulin (*μ*UI/mL)	−0.350**
HOMA_IR_	−0.344**
Triglycerides (mg/mL)	−0.343**
HDL cholesterol (mg/mL)	−0.016
Total cholesterol (mg/mL)	−0.159
CRP (mg/dL)	−0.320**
C_3_ (g/L)	−0.258*
C_4_ (g/L)	−0.302*

HOMA_IR_: homeostasis model assessment.

*P < 0.05, **P < 0.01.

## References

[1] Holick MF (2007). Medical progress: vitamin D deficiency. *The New England Journal of Medicine*.

[2] Dawson-Hughes B (2008). Serum 25-hydroxyvitamin D and functional outcomes in the elderly. *American Journal of Clinical Nutrition*.

[3] van Schoor NM, Visser M, Pluijm SMF, Kuchuk N, Smit JH, Lips P (2008). Vitamin D deficiency as a risk factor for osteoporotic fractures. *Bone*.

[4] Nagpal S, Na S, Rathnachalam R (2005). Noncalcemic actions of vitamin D receptor ligands. *Endocrine Reviews*.

[5] Rosen CJ (2011). Vitamin D insufficiency. *The New England Journal of Medicine*.

[6] Parikh SJ, Edelman M, Uwaifo GI (2004). The relationship between obesity and serum 1,25-dihydroxy vitamin D concentrations in healthy adults. *Journal of Clinical Endocrinology and Metabolism*.

[7] Cheng S, Massaro JM, Fox CS (2010). Adiposity, cardiometabolic risk, and vitamin D status: the framingham heart study. *Diabetes*.

[8] Need AG, O’Loughlin PD, Horowitz M, Nordin BEC (2005). Relationship between fasting serum glucose, age, body mass index and serum 25 hydroxyvitamin D in postmenopausal women. *Clinical Endocrinology*.

[9] Konradsen S, Ag H, Lindberg F, Hexeberg S, Jorde R (2008). Serum 1,25-dihydroxy vitamin D is inversely associated with body mass index. *European Journal of Nutrition*.

[10] Liu E, Meigs JB, Pittas AG (2010). Predicted 25-hydroxyvitamin D score and incident type 2 diabetes in the Framingham Offspring Study. *American Journal of Clinical Nutrition*.

[11] Scragg R, Sowers M, Bell C (2004). Serum 25-hydroxyvitamin D, diabetes, and ethnicity in the Third National Health and Nutrition Examination Survey. *Diabetes Care*.

[12] Lu L, Yu Z, Pan A (2009). Plasma 25-hydroxyvitamin D concentration and metabolic syndrome among middle-aged and elderly Chinese individuals. *Diabetes Care*.

[13] Zhao G, Ford ES, Li C (2010). Associations of serum concentrations of 25-hydroxyvitamin D and parathyroid hormone with surrogate markers of insulin resistance among U.S. adults without physician-diagnosed diabetes: NHANES, 2003–2006. *Diabetes Care*.

[14] Liu E, Meigs JB, Pittas AG (2009). Plasma 25-hydroxyvitamin D is associated with markers of the insulin resistant phenotype in nondiabetic adults. *Journal of Nutrition*.

[15] Chiu KC, Chu A, Go VLW, Saad MF (2004). Hypovitaminosis D is associated with insulin resistance and *β* cell dysfunction. *American Journal of Clinical Nutrition*.

[16] Jablonski KL, Chonchol M, Pierce GL, Walker AE, Seals DR (2011). 25-Hydroxyvitamin D deficiency is associated with inflammation-linked vascular endothelial dysfunction in middle-aged and older adults. *Hypertension*.

[17] Peterson CA, Heffernan ME (2008). Serum tumor necrosis factor-alpha concentrations are negatively correlated with serum 25(OH)D concentrations in healthy women. *Journal of Inflammation*.

[18] Ngo DT, Sverdlov AL, McNeil JJ, Horowitz JD (2010). Does vitamin D modulate asymmetric dimethylarginine and C-reactive protein concentrations?. *American Journal of Medicine*.

[19] Dobnig H, Pilz S, Scharnagl H (2008). Independent association of low serum 25-hydroxyvitamin D and 1,25-dihydroxyvitamin D levels with all-cause and cardiovascular mortality. *Archives of Internal Medicine*.

[20] Bellia A, Garcovich C, D’Adamo M Serum 25-hydroxyvitamin D levels are inversely associated with systemic inflammation in severe obese subjects.

[21] Hyppönen E, Berry D, Cortina-Borja M, Power C (2010). 25-Hydroxyvitamin D and pre-clinical alterations in inflammatory and hemostatic markers: a cross sectional analysis in the 1958 British Birth Cohort. *PloS one*.

[22] Peterson CA, Heffernan ME (2008). Serum tumor necrosis factor-alpha concentrations are negatively correlated with serum 25(OH)D concentrations in healthy women. *Journal of Inflammation*.

[23] Vilarrasa N, Vendrell J, Maravall J (2010). Is plasma 25(OH) D related to adipokines, inflammatory cytokines and insulin resistance in both a healthy and morbidly obese population?. *Endocrine*.

[24] Zittermann A, Frisch S, Berthold HK (2009). Vitamin D supplementation enhances the beneficial effects of weight loss on cardiovascular disease risk markers. *American Journal of Clinical Nutrition*.

[25] Amer M, Qayyum R (2012). Relation between serum 25-hydroxyvitamin D and C-reactive protein in asymptomatic adults (from the continuous National Health and Nutrition Examination Survey 2001 to 2006). *American Journal of Cardiology*.

[26] Mohamed-Ali V, Goodrick S, Rawesh A (1997). Subcutaneous adipose tissue releases interleukin-6, but not tumor necrosis factor-*α*, in vivo. *Journal of Clinical Endocrinology and Metabolism*.

[27] Gabrielsson BG, Johansson JM, Lönn M (2003). High expression of complement components in omental adipose tissue in obese men. *Obesity Research*.

[28] Koistinen HA, Vidal H, Karonen SL (2001). Plasma acylation stimulating protein concentration and subcutaneous adipose tissue C3 mRNA expression in nondiabetic and type 2 diabetic men. *Arteriosclerosis, Thrombosis, and Vascular Biology*.

[29] Muscari A, Massarelli G, Bastagli L (2000). Relationship of serum C3 to fasting insulin, risk factors and previous ischaemic events in middle-aged men. *European Heart Journal*.

[30] Muscari A, Antonelli S, Bianchi G (2007). Serum C3 is a stronger inflammatory marker of insulin resistance than C-reactive protein, leukocyte count, and erythrocyte sedimentation rate: Comparison study in an elderly population. *Diabetes Care*.

[31] De Pergola G, Ciccone MM, Guida P (2011). Relationship between C3 levels and common carotid intima-media thickness in overweight and obese patients. *Obesity Facts*.

[32] Pearson TA, Mensah GA, Alexander RW (2003). Markers of inflammation and cardiovascular disease: Application to clinical and public health practice: a statement for healthcare professionals from the centers for disease control and prevention and the American Heart Association. *Circulation*.

[33] Bonora E, Targher G, Alberiche M (2000). Homeostasis model assessment closely mirrors the glucose clamp technique in the assessment of insulin sensitivity: studies in subjects with various degrees of glucose tolerance and insulin sensitivity. *Diabetes Care*.

[34] Wortsman J, Matsuoka LY, Chen TC, Lu Z, Holick MF (2000). Decreased bioavailability of vitamin D in obesity. *American Journal of Clinical Nutrition*.

[35] Tzotzas T, Papadopoulou FG, Tziomalos K (2010). Rising serum 25-hydroxy-vitamin D levels after weight loss in obese women correlate with improvement in insulin resistance. *Journal of Clinical Endocrinology and Metabolism*.

[36] Shahar DR, Schwarzfuchs D, Fraser D (2010). Dairy calcium intake, serum vitamin D, and successful weight loss. *American Journal of Clinical Nutrition*.

[37] Ding C, Parameswaran V, Blizzard L, Burgess J, Jones G (2010). Not a simple fat-soluble vitamin: changes in serum 25-(OH)D levels are predicted by adiposity and adipocytokines in older adults. *Journal of Internal Medicine*.

[38] De Pergola G, Ammirati A, Caccavo D, Bavaro S, Barone G (2012). Vitamin D, obesity and risk of diabetes. *Nutritional Therapy and Metabolism*.

[39] Boucher BJ, Mannan N, Noonan K, Hales CN, Evans SJW (1995). Glucose intolerance and impairment of insulin secretion in relation to vitamin D deficiency in East London Asians. *Diabetologia*.

[40] Kayaniyil S, Vieth R, Retnakaran R (2010). Association of vitamin D with insulin resistance and *β*-cell dysfunction in subjects at risk for type 2 diabetes. *Diabetes Care*.

[41] Borissova AM, Tankova T, Kirilov G, Dakovska L, Kovacheva R (2003). The effect of vitamin D3 on insulin secretion and peripheral insulin sensitivity in type 2 diabetic patients. *International Journal of Clinical Practice*.

[42] Nagpal J, Pande JN, Bhartia A (2009). A double-blind, randomized, placebo-controlled trial of the short-term effect of vitamin D3 supplementation on insulin sensitivity in apparently healthy, middle-aged, centrally obese men. *Diabetic Medicine*.

[43] Von Hurst PR, Stonehouse W, Coad J (2010). Vitamin D supplementation reduces insulin resistance in South Asian women living in New Zealand who are insulin resistant and vitamin D deficient-a randomised, placebo-controlled trial. *British Journal of Nutrition*.

[44] Maestro B, Campión J, Dávila N, Calle C (2000). Stimulation by 1,25-dihydroxyvitamin D3 of insulin receptor expression and insulin responsiveness for glucose transport in U-937 human promonocytic cells. *Endocrine Journal*.

